# The Spectrum of Central Nervous System Infections in an Adult Referral Hospital in Hanoi, Vietnam

**DOI:** 10.1371/journal.pone.0042099

**Published:** 2012-08-30

**Authors:** Walter R. Taylor, Kinh Nguyen, Duc Nguyen, Huyen Nguyen, Peter Horby, Ha L. Nguyen, Trinh Lien, Giang Tran, Ninh Tran, Ha M. Nguyen, Thai Nguyen, Ha H. Nguyen, Thanh Nguyen, Giap Tran, Jeremy Farrar, Menno de Jong, Constance Schultsz, Huong Tran, Diep Nguyen, Bich Vu, Hoa Le, Trinh Dao, Trung Nguyen, Heiman Wertheim

**Affiliations:** 1 Oxford University Clinical Research Unit, Hanoi, Vietnam; 2 Centre for Vaccinology and Tropical Medicine, Churchill Hospital, Oxford, United Kingdom; 3 National Hospital for Tropical Diseases, Hanoi, Vietnam; 4 Oxford University Clinical Research Unit, Ho Chi Minh City, Vietnam; Pennsylvania State University College of Medicine, United States of America

## Abstract

**Objectives:**

To determine prospectively the causative pathogens of central nervous system (CNS) infections in patients admitted to a tertiary referral hospital in Hanoi, Vietnam.

**Methods:**

From May 2007 to December 2008, cerebrospinal fluid (CSF) samples from 352 adults with suspected meningitis or encephalitis underwent routine testing, staining (Gram, Ziehl-Nielsen, India ink), bacterial culture and polymerase chain reaction targeting *Neisseria meningitidis*, *Streptococcus pneumoniae, S. suis, Haemophilus influenzae* type b, *Herpes simplex* virus (HSV), Varicella Zoster virus (VZV), enterovirus, and 16S ribosomal RNA. Blood cultures and clinically indicated radiology were also performed. Patients were classified as having confirmed or suspected bacterial (BM), tuberculous (TBM), cryptococcal (CRM), eosinophilic (EOM) meningitis, aseptic encephalitis/meningitis (AEM), neurocysticercosis and others.

**Results:**

352 (male: 66%) patients were recruited: median age 34 years (range 13–85). 95/352 (27.3%) diagnoses were laboratory confirmed and one by cranial radiology: BM (n = 62), TBM (n = 9), AEM (n = 19), CRM (n = 5), and neurocysticercosis (n = 1, cranial radiology). *S. suis* predominated as the cause of BM [48/62 (77.4%)]; *Listeria monocytogenese* (n = 1), *S. pasteurianus* (n = 1) and *N. meningitidis* (n = 2) were infrequent. AEM viruses were: HSV (n = 12), VZV (n = 5) and enterovirus (n = 2). 5 patients had EOM. Of 262/352 (74.4%) patients with full clinical data, 209 (79.8%) were hospital referrals and 186 (71%) had been on antimicrobials. 21 (8%) patients died: TBM (15.2%), AEM (10%), and BM (2.8%).

**Conclusions:**

Most infections lacked microbiological confirmation. *S. suis* was the most common cause of BM in this setting. Improved diagnostics are needed for meningoencephalitic syndromes to inform treatment and prevention strategies.

## Introduction

Central nervous system (CNS) infections encompass a wide range of pathogens and are an important cause of morbidity and mortality worldwide. [1]–[4]

In Vietnam, surveillance for CNS infections by the public health system is based on clinical definitions. Most aetiological data have come from recent studies conducted at referral hospitals in Ho Chi Minh City, in southern Vietnam. [5]–[8] These series have identified *Streptococcus suis* as the leading cause of bacterial meningitis (BM), accounting for ∼40% of BM cases. Other bacteria were *S. pneumoniae* (∼23%), *N. meningitidis* (∼8%), *Klebsiella pneumoniae* (∼3%), and ∼2.5% each for *Escherichia coli, Haemophilus influenzae* and *Staphylococcus aureus*. [9] Recently, three adults were diagnosed with cerebrospinal fluid culture (CSF) culture positive *Listeria monocytogenes* meningitis, a very rare cause of meningitis in Vietnam. [10] Older data from Vietnam have documented *N. meningitidis* [11], *Yersinia pestis* [12] and *Pseudomonas pseudomallei* [13] as causes of meningitis.

Japanese B encephalitis virus (JEV), an important cause of acute paediatric encephalitis and was the most commonly detected virus (26%) in a prospective series of 194 children. [14] Other isolated viruses were enteroviruses (9.3%), dengue (4.6%) and 0.5% each for *Herpes simplex*, Cytomegalovirus and influenza A/H5N1. More than half of these patients lacked a confirmed diagnosis.

Accurate estimates of the contribution of meningitis due to *Mycobacterium tuberculosis* (TBM) are hampered by the difficulty of confirming the diagnosis. In HCM, TBM is thought to account for about one third of admitted meningitis patients. [15] A diagnostic algorithm, developed by data from HCM, can distinguish TB from bacterial meningitis with a high degree of confidence using clinical and laboratory criteria. [7]

Eosinophilic meningitis is uncommon in Vietnam [15], often lacks an etiological diagnosis and is treated empirically with albendazole and corticosteroids. Several parasites that can invade the CNS are endemic in Vietnam, including *Angiostrongylus cantonensis* [16], *Gnathostoma spinerigium* [17], *Trichinella spiralis* [18], cysticercosis [19], lung fluke [20] and toxocara. [21]

In northern Vietnam, CNS infection data are limited. Like in southern Vietnam, *S. suis* is an important cause of bacterial meningitis in patients presenting in Hanoi. [22] One study identified JE as a commonly diagnosed cause of acute paediatric encephalitis but was rare in adults. [23] To date, no prospective study examining the microbiological aetiologies of meningitis or encephalitis in adults has been conducted in Hanoi. Herein, we report the findings of such a study.

## Methods

### Study design and site

This prospective study examining the causes of CNS infections in hospitalized patients took place from May 2007 to December 2008 at the National Hospital for Tropical Diseases (NHTD) in Hanoi, the capital of Vietnam. NHTD is an adult tertiary referral, infectious diseases hospital covering Hanoi and the northern provinces. It has 150 beds and approximately 4,000 admissions per year.

The climate in Vietnam is subtropical in the northern areas and tropical in the southern areas. The rainy season affects the whole country from May to September. Thereafter, in northern Vietnam, the climate is cool with cloudy days and occasional light rain. The winter months can be cold and temperatures can be as low as 6 to 10°C (http://www.vietnamembassy.org.uk/climate.html, accessed October 2010). The study was approved by the Scientific and Ethical Committee of the NHTD and the Oxford University Tropical Ethics Committee (OxTREC).

### Patients and study conduct

Patients were eligible for the study if they had clinical evidence of a CNS infection, based on the judgment of the admitting doctor, and they or a legal guardian provided written informed consent. Exclusion criteria included: (i) patients with a known pre-existing neurological condition e.g. cerebral tumour, receiving antibiotics for a cerebral abscess, cerebrospinal fluid (CSF) shunt *in situ*, and neurosurgery within the previous two months.

Patients were managed by hospital doctors following routine clinical practice i.e. history, physical examination, haematology, biochemistry, blood culture, and lumbar punctures (LP), and radiology. The research team supplied LP manometers to measure CSF opening pressures, ophthalmoscopes and tuning forks (Rinné and Weber tests). HIV testing was not part of the study protocol but was done as clinically indicated. The study protocol mandated, *inter alia*, the taking of a blood culture on admission, and a preLP cranial CT scan if there was evidence of raised intracranial pressure or a focal neurological sign.

At NHTD, acute bacterial meningitis (BM) is generally treated with a combination of high dose intravenous (IV) ceftriaxone (2 g bd) and four days of high dose steroids. [24] Patients older than 50 years with bacterial meningitis are treated with ceftriaxone in combination with ampicillin. TBM is treated with rifampicin, isoniazid, pyrazinamide, ethambutol, and streptomycin together with steroids, consistent with local and international guidelines. [25], [26] Post discharge, TB patients are cared for by the TB service. Suspected or confirmed *Herpes simplex* (HSV) and varicella zoster virus (VZV) encephalitis are treated with acyclovir.

### Diagnostic microbiology

The NHTD clinical microbiology laboratory provides a diagnostic service that includes automated bacterial blood culture [Bactec™ Beckton Dickinson (BD), USA], standard media for bacterial and fungal cultures, microscopy (Gram and Ziehl-Nielsen [ZN] stains), *Mycobacterium tuberculosis* culture (MGIT™, Becton Dickinson, USA).

The Oxford University Clinical Research Unit (OUCRU) was set up in 2006 and established a research molecular laboratory, performing PCR for several viruses and bacteria and a genome sequencing facility for 16S ribosomal (r) RNA analysis. The molecular diagnostic laboratory has separated laboratories for the different steps for PCR and has a unidirectional work flow to prevent contamination. The hospital laboratories participate in an external quality assurance scheme.

Physicians were encouraged to take at least 5 mL of CSF. All CSF specimens were processed immediately for testing or storage at −80°C for later analysis. Testing was done in batches at least once per week. Routine CSF testing involved: (i) Gram stain for bacteria, (ii) ZN stain for mycobacteria/acid fast bacilli (AFB), (iii) India ink stain in HIV positive patients for suspected *Cryptococcus neoformans*, (iv) bacterial culture, (v) glucose and total protein, and (vi) total and differential white cell count (WCC). Further bacterial identification was done according to standard microbiologic techniques, including biochemical tests (API® strips, Biomerieux, France). Mycobacterial culture was only done if the treating physician requested it for patients with suspected TBM.

PCR tests were done according to previously published methods for selected bacteria and viruses, with the following targets: (i) *Streptococcus suis* [5], (ii) *S. pneumoniae* [27], (iii) *Neisseria meningitidis* [27], (iv) *Haemophilus influenza* type b [27]. The viruses tested were: (i) enterovirus (EV) [28], (ii) HSV 1 and 2 [29], and (iii) VZV [30]. Subsequently, selected negative CSF samples were tested for (i) Nipah virus (n = 81) in patients with non-purulent meningitis [31], and (ii) bacteria by 16S rRNA in patients with suspected bacterial meningitis (n = 63). [32]

### Definitions

A microbiologically confirmed diagnosis was one in which any of the microbiological investigations (i.e. stain, culture, PCR) was positive for a pathogen that was consistent with the clinical picture. If patients had evidence of more than one CNS pathogen, the clinical diagnosis was made according to the dominant clinical picture and CSF findings. *Post hoc*, a diagnostic cranial CT scan for neurocysticercosis was considered sufficient to confirm that diagnosis.

Diagnostic categories were: bacterial (BM), tuberculous (TBM), cryptococcal (CRM), eosinophilic (EOM) meningitis, aseptic encephalitis/meningitis (AEM), neurocysticercosis and miscellaneous. An eosinophilic meningitis of presumed parasitic aetiology was diagnosed if the CSF contained >10 eosinophils/mm3 and/or eosinophils accounting for >10 percent of CSF leukocytes. Clinically suspected diagnoses were made by the treating physicians based on all the clinical information i.e. clinical picture, CSF findings, radiology (chest×rays, brain imaging), and the response to treatment.

Disability was assessed using the Modified Rankin score (MRS) from 0 to 6 [33]: (i) 0 = no symptoms, (ii) 1 = no significant disability despite symptoms; able to carry out all usual duties and activities, (iii) 2 = slight disability; unable to carry out all previous activities, but able to look after own affairs without assistance, (iv) 3 = moderate disability; requiring some help, but able to walk without assistance, (v) 4 = moderately severe disability; unable to walk without assistance and unable to attend to own bodily needs without assistance, (vi) 5 = severe disability; bedridden, incontinent and requiring constant nursing care and attention, and (vii) 6 = Dead.

### Data analysis

Confirmed diagnoses are based on all 352 patients; of these, 262 patients had full clinical data.

Analyses were descriptive: (i) chi squared or Fisher's exact test for proportional data, and (ii) Kruskall Wallis or the ranksum test for continuous data and done between (a) patients with a confirmed (c) vs. a clinically suspected (s) diagnosis (Table 1), and (b) the three main diagnostic groups by combining the confirmed and suspected diagnoses (Table 2). A p value ≤0.05 was considered statistically significant. No adjustments were made for multiple comparisons.

**Table 1:** Patient demographic, clinical and laboratory features diagnosed with confirmed or clinically suspected bacterial meningitis, tuberculous meningitis and aseptic encephalitis/meningitis.

	Bacterial meningitis		P	Tuberculous meningitis		P	Aseptic encephalitis meningitis		P
	confirmed n = 62	Suspected n = 51		confirmed n = 9	suspected n = 58		confirmed n = 19	suspected n = 54	
***Demographic & general data***									
Age	46 (17–78)	40 (14–76)	0.34	36 (15–69)	34 (14–76)	0.6	30 (17–85)	26 (13–75)	0.052
F∶M	10∶52	18∶33	0.019	4∶5	19∶39	0.49	7∶12	29 25	0.2
HIV positive by history	0/58 (0)	2 (3.9)	0.12	3/8 (37.5)	13 (22.4)	0.35	0/16 (0)	2 (3.7)	0.43
Referred by another hospital	49/58 (84.5)	44 (86.2)	0.79	5/8 (62.5)	43 (74.1)	0.48	12/16 (75)	43 (79.6)	0.69
Preadmission antibiotic use	39/58 (67.2)	44 (86.3)	0.02	5/8 (62.5)	40 (68.9)	0.71	11/16 (68.75)	36 (66.7)	0.87
Length of illness	3.5 (0–17)	6.5 (1–31)	0.34	12.5 (1–31)	15.5 (2–61)	0.61	7 (0–22)	6 (1–31)	0.051
***Symptoms***									
Fever	58/58 (100)	50 (98.1)	0.28	7/8 (87.5)	57 (98.3)	0.096	16/16 (100)	51 (94.4)	0.33
Headache	54/58 (93.1)	51 (100)	0.056	8/8 (100)	58 (100)	-	14/16 (87.5)	52 (96.3)	0.18
Vomiting	45 (77.6)	43 (84.3)	0.37	5/8 (62.5)	39 (67.2)	0.79	8/16 (50)	34 (62.9)	0.35
Convulsions	5/58 (8.6)	6 (11.7)	0.58	0 (0)	4 (6.9)	0.4	5/16 (31.25)	17 (31.5)	0.98
***Signs***									
Temperature °C	38.5 (36.4–40)	38 (36–39.8)	0.003	37.2 (36.2–38)	37.8 (36–39.9)	0.054	38.4 (37–39.8)	38.2 (36.5–40.5)	0.73
GCS	13 (6–15)	15 (5–15)	0.006	14 (11–15)	15 (6–15)	0.54	13 (5–15)	13 (4–15)	0.56
GCS = 15	18/57 (31.6)	28/49 (57.1)	0.008	3/8 (37.5)	32 (55.2)	0.34	7/16 (43.75)	15/54 (27.8)	0.22
Stiff neck	55 (94.8)	41 (80.4)	0.02	8 (100)	48 (82.7)	0.2	14 (87.5)	42 (77.8)	0.39
Kernig's sign	50 (86.2)	28 (54.9)	0.00	6 (75)	40 (68.9)	0.72	8 (50)	35 (64.8)	0.28
Skin rash	6/58 (10.3)	5/51 (9.8)	0.9	0 (0)	2 (3.45)	0.59	1/16 (6.25)	3/54 (5.6)	0.9
***Routine haematology***									
Haemoglobin g/dL	12.9 (8.5–15.7)	12.6 (8.7–16.6)	0.32	11.3 (10–13)	12.4 (3.75–16.7)	0.17	12.7 (10.1–15.6)	13 (5.6–17.9)	0.9
Total white cell count/µL	15.5 (6.28–36.2)	12.3 (3.27–29.36)	0.0001	9.3 (2–16)	9.13 (1.73–28.8)	0.91	9.96 (5.6–23.7)	10 (3.5–25.9)	0.77
Leukocytosis >11000/µL	51/60 (85)	29 (56.8)	0.001	4 (44.4)	19 (32.7)	0.49	4/17 (23.5)	23 (42.6)	0.15
Relative neutrophilia >75%	54/59 (81.3)	26/50 (52)	0.000	9 (100)	34/57 (59.6)	0.018	10/16 (62.5)	31/52 (59.6)	0.83
Platelet count/µL	126 (4–770)	236 (21–490)	0.000	250 (112–587)	248 (72–456)	0.78	209 (4.51–369)	208 (54–628)	0.88
***Lumbar puncture data***									
Illness duration at LP	4 (0–17)	7 (1–31)	0.0007	12.5 (1–31)	15.5 (3–61)	0.29	7.5 (0–22)	6 (1–32)	0.45
Cloudy	45/55 (81.8)	19/48 (39.6)	0.000	6/8 (75)	17/54 (31.5)	0.017	4/16 (25)	12/53 (22.64)	0.84
Opening pressure cm CSF	27 (6–40)	20 (7–40)	0.037	20 (7.5–37)	26.25 (5–40)	0.47	17.25 (7–29)	11.5 (5–40)	0.8
Opening pressure >20 cm CSF	21/29 (72.4)	17/36 (47.2)	0.04	3/6 (50)	22/34 (64.7)	0.49	2/8 (25)	10/37 (27.1)	0.9
Total white cell count/mm^3^	1750 (10–34,000)	195 (10–42,900)	0.000	391 (67–4300)	215 (8–1300)	0.2	115 (5–600)	54 (2–770)	0.06
% >50% neutrophilia	49/59 (83.1)	19/46 (41.3)	0.000	4/9 (44.4)	10/53 (18.8)	0.09	1/16 (6.25)	3/36 (8.3)	0.79
Glucose mmol/L	1.1 (0.05–4.8)	2.6 (0.4–6.6)	0.000	1.2 (0.5–2.7)	1.9 (0.2–8.2)	0.05	3.3 (1.2–5.8)	3.45 (1.76–6.6)	0.89
CSF∶blood glucose ratio	0.17 (0.009–1.1)	0.4 (0.059–1.06)	0.000	0.21 (0.08–0.41)	0.29 (0.075–1)	0.01	0.5 (0.19–0.92)	0.53 (0.21–0.8)	0.72
CSF∶blood ratio <50%	38/45 (84.4)	32/41 (78.05)	0.44	7/7 (100)	37/47 (78.7)	0.18	6/14 (42.8)	16/38 (42.1)	0.96
Total protein g/L	1.85 (0.56–7.5)	0.82 (0.33–3.75)	0.000	1.6 (0.5–2.3)	1.4 (0.39–11.8)	0.61	1.25 (0.2–4.2)	0.7 (0.13–2.4)	0.27

All continuous data are median (range).

**Table 2:** Selected patient characteristics when confirmed and clinically suspected diagnoses are combined.

	Bacterial meningitis n = 113	Tuberculous meningitis n = 67	Aseptic encephalitis meningitis n = 73	P for 3 group comparisons
***Symptoms***				
Convulsions	11/109 (10.1)	4/66 (6.1)	22/70 (31.4)	0.000*
***Routine haematology***				
Total white cell count ×1000/µL	13.9 (3.27–36.2)	9.17 (1.73–28.8)	9.96 (3.3–29.96)	0.0001†
Leukocytosis >11,000/µL	80/111 (72.1)	23/67 (34.3)	27/71 (38.03)	0.000†
Neutrophilia ≥75%	80/109 (73.4)	43/66 (65.1)	41/68 (60.3)	0.17
***Lumbar puncture data***				
Cloudy	64/103 (62.1)	23/62 (37.1)	16/69 (23.2)	0.000#
Opening pressure cm CSF	24 (6–40) n = 65	25 (5–40) n = 40	17 (5–40) n = 45	0.00048*
Opening pressure >20 cm CSF	38/65 (58.4%)	25/40 (62.5%)	12/45 (26.6)	0.001‡
Total white cell count/µL	500 (10–42,900)	220 (8–4300)	63 (2–770)	0.0001§
% >50% neutrophilia	68/105 (64.8)	14/62 (22.6%)	4/52 (7.7)	0.000†
CSF∶blood glucose ratio	0.27 (0.009–1.1)	0.27 (0.075–1)	0.52 (0.19–0.92)	0.00018*
CSF∶blood glucose ratio <50%	70/86 (81.4)	44/54 (81.4)	22/52 (42.3)	0.000*
Total protein g/L	1.39 (0.33–7.5)	1.4 (0.39–11.8)	0.7 (0.13–4.2)	0.0001*

All continuous data are median (range).

* AEM vs. TBM & AEM vs. BM, ps≤0.000.

† BM vs. TBM & BM vs. AEM, ps = 0.000.

# BM vs. TBM p = 0.002, BM vs. AEM, p = 0.000.

‡ AEM vs. BM & TBM, ps 0.001.

§ AEM vs. BM & TBM, ps<0.000, TBM vs. AEM, p = 0.018.

## Results

**Figure 1:**
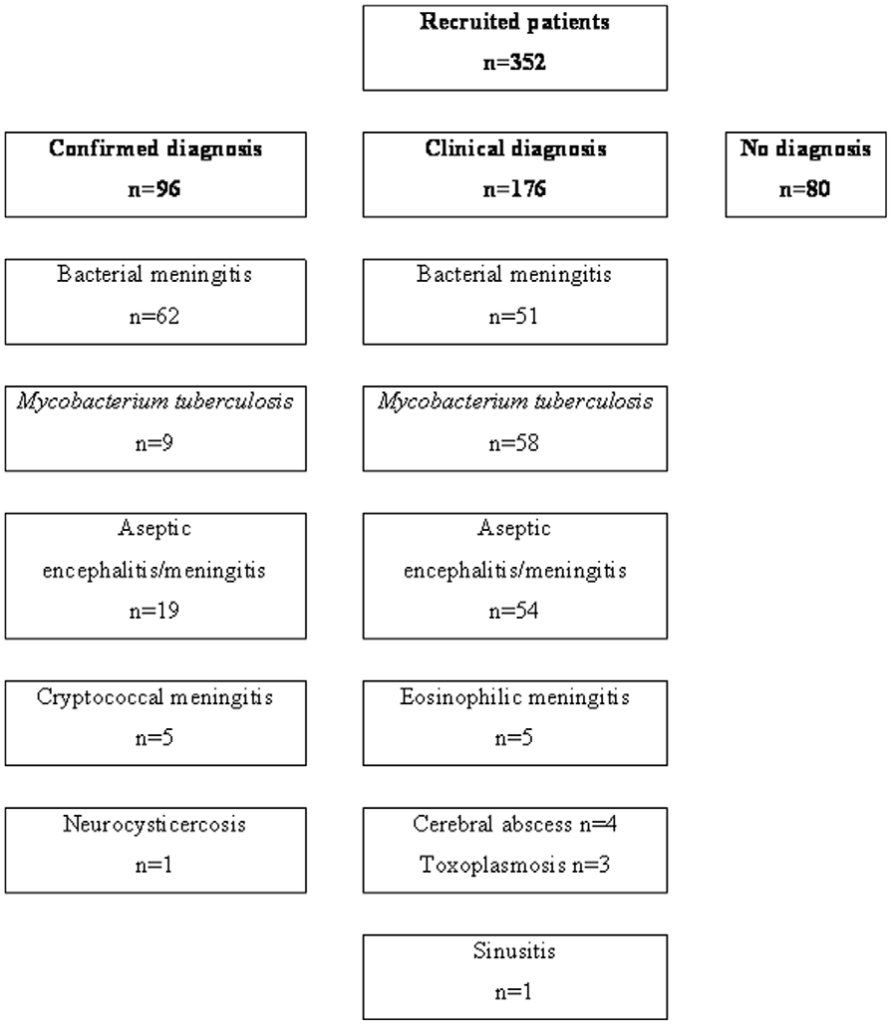
Profile of patient numbers with confirmed and clinically diagnosed meningitis or encephalitis.

A confirmed etiological diagnosis (Figure 1) was made in 96 of 352 (27.3%) patients. The three main confirmed diagnoses were cBM, 62/96 (64.6%), followed by cAEM, 19/96 (19.8%), and cTBM, 9/96 (9.4%). Detailed data are presented for these three groups.

### General characteristics

The 262 patients had a median age of 34.5 (range 13–85) years; 92 (35.1%) were females. 100 (38.2%) patients (72 male, 28 female) had a potential, occupational exposure pigs or pork; 95 were farmers (37.1%), 3 butchers, one each a cook and pig seller. This potential occupational exposure was significant (p = 0.01) in the cBM 32/55 (58.2%) vs. sBM 17/51 (33.3%) patients and (p = 0.028) in the combined (com) BM [49/106 (46.2%)] patients compared to the comTBM patients [19/65 (29.2%)].

Patients were drawn from 29 provinces. Hanoi province (Hanoi and its conurbation) had the highest number of patients, 35/259 (13.5%) but this was not associated with a particular diagnosis (data not shown). Seasonality was not observed (data not shown).

### Clinical data

Preadmission illnesses lasted a median of 7 days, range 0 to 92. The majority of patients, 209/262 (79.8%), were referred from another hospital and 186/262 (71%) were already taking antimicrobial drugs: 4 on oral acyclovir, 6 anti-TB treatment and 176 on antibiotics. 27 patients reported they were HIV positive of whom 16 were in the comTBM group (3 confirmed, 13 suspected).

Overall, fever (252/262, 96.9%) and headache (252/262, 96.2%) were the most commonly recorded symptoms. Neck pain (36.2%, n = 95), convulsions (14.1%, n = 37), and reduced hearing (8.8%, n = 23) were reported less frequently. Photophobia (4.2%, n = 11) was uncommon.

Median vital signs parameters were within normal limits and similar between the groups (data not shown). 116/259 (44.8%) patients had a normal Glasgow Coma Score (GCS) of 15; 22 (8.5%) had unrousable coma, GCS ≤8. A stiff neck (218/262, 83.2%) and Kernig's sign (175/262, 66.8%) were common. A minority of patients had neurological signs e.g. oculomotor (n = 5) and abducens (n = 2) nerve palsies, hemiparesis (n = 12), monoparesis (n = 5), or paraplegia (n = 3). Profound hearing loss (inability to hear tuning fork) was detected in 12 patients, 7 on admission and in 5 new patients by discharge. Their diagnoses were: 7 *S. suis*, 1 *S. pneumoniae*, 3 sBM and 1 with cTBM.

Comparing confirmed with suspected diagnoses revealed several important, statistically significant differences. For patients with cBM (vs. sBM), males were more frequently affected, the median GCS and proportion with a normal GCS were less, meningeal signs more common and they had more frequent blood leukocytosis and relative neutrophilia ≥75%. The cTB patients were more likely to have a blood relative neutrophilia compared to patients with sTBM. There were no outstanding differences between the cAEM and sAEM groups but the comAEM group was most likely to have had convulsions (Table 2).

### Lumbar puncture data

The majority of patients, 247/262 (90.5%), had LPs done either on admission (n = 208, 79.4%) or by the next day (n = 29), on median illness day 8 (range 0 to 100). Opening pressures were raised (>20 cm CSF) in 82 of 163 (50.3%) patients.

Relative to the sBM group, the confirmed BM group had LPs done sooner and all but one of the CSF findings were significantly different and more typical of an untreated BM. The cTBM group had more cloudy CSFs and a lower median CSF∶blood glucose ratio, compared to the sTBM patients. There were no significantly different CSF findings between the two AEM groups but the median total CSF WCC tended to be higher in the confirmed group.

Comparing the combined groups, certain CSF findings are similar between the BM and TBM groups, notably, CSF opening pressure, glycaemic parameters and the total protein. The AEM group has the lowest median opening CSF pressure, median CSF WCC, median total protein but highest median CSF∶blood glucose ratio and the lowest proportion of patients with a CSF∶blood glucose ratio <50%.

### Microbiology

*S. suis* (n = 48) was the most commonly identified bacterium in the BM group, followed by *S. pneumoniae* (n = 7). *N. meningitidis* (n = 2), *L. monocytogenes* and *S. pasteurianus* (Table 3). HSV (n = 12) and VZV (n = 5) were the most commonly detected viruses in the AEM group; EV was detected in 2 patients. *M. tuberculosis* was confirmed in 9 patients. PCR had an important impact on diagnostic confirmation (Table 3). Standard staining of CSF detected: (i) Gram positive cocci in 22 of 53 (41.5%) cBM and 23 of 94 (24.5%) cBM patients (Gram stain), (ii) no patients with acid fast bacilli (ZN stain), and (ii) 5 patients with cryptococcus (India ink).

**Table 3:** Pathogens isolated or detected by routine culture and or molecular methods.

	Total N confirmed	Blood culture positive	CSF culture positive	PCR positive	Molecular alone*	16S RNA	Blood culture alone	CSF culture alone	Blood & CSF culture & PCR positive	CSF culture & PCR positive	Mixed results†
*S. suis*	48	10	27	43	19 (39.6)	0	1	3	7	16	2
*S. pneumoniae*	7	1	3	6	3 (42.9)	1	0	0	4	0	NA
*Neisseria meningitidis*	2	0	0	2	2 (100)	0	0	0	0	0	NA
*S. pasteurianus*	1	0	0	NA	1 (100)	1	0	0	0	0	NA
*Klebsiella pneumoniae*	1	0	0	NA	1 (100)	1	0	0	0	0	NA
*Listeria monocytogenese*	1	0	0	NA	1 (100)	1	0	0	0	0	NA
*S. aureus*	1	1	0	NA	0	0	1	0	0	0	NA
Streptococcus species	1	1	1	0	0	0	1	0	0	0	NA
*H. simplex*	12	NA	NA	12	12 (100)	NA	NA	NA	NA	NA	NA
Enterovirus (generic)	2	NA	NA	2	2 (100)	NA	NA	NA	NA	NA	NA
Varicella zoster virus	5	NA	NA	5	5 (100)	NA	NA	NA	NA	NA	NA
*M. tuberculosis*	9	NA	4	5	3 (33.3)	NA	NA	4	NA	2	NA

* PCR or 16S rRNA. Proportion of total confirmed diagnoses due to molecular detection.

† blood culture & PCR positive, blood & CSF culture positive, PCR not done.

A history of preadmission antibiotic use did not affect significantly (p = 0.4) the Gram stain positivity rate in the cBM group: 37.8% (14/37) vs. 50% (8/16 not on antibiotics) but did (p = 0.02) for a confirmed diagnosis of BM: 39/83 (46.9% on antibiotics) vs. 19/26 (73.1% not on antibiotics).

### Outcomes

There were 21/262 (8%) deaths, 20 in the main 3 groups (Table 4) and one death in a patient with suspected cerebral toxoplasmosis. Most deaths (15.5%) occurred in the sTBM group which also had a significantly shorter, median hospital admission time compared to the cTBM group.

**Table 4:** Patient outcomes.

	Bacterial meningitis		P	Tuberculous meningitis		P	Aseptic encephalitis/meningitis		P
	confirmed n = 57	suspected n = 51		Confirmed n = 9	suspected n = 58		confirmed n = 16	suspected n = 53	
Inpatients days†	15.5 (2–48)	14.5 (3–44)	0.29	27.5 (7–42)	14 (0–69)	0.018	14.5 (4–59)	11 (0–39)	0.34
Modified Rankin score									
0	46 (80.7)	44 (86.3)	0.53*	7 (87.5)	43 (74.1)	0.9*	8 (50)	35 (66.1)	0.45*
1	6 (10.5)	2 (3.9)		0 (0)	2 (3.45)		1 (6.25)	4 (7.5)	
2	0 (0)	2 (3.9)		0 (0)	1 (1.7)		0 (0)	2 (3.8)	
3	1 (1.75)	0 (0)		0 (0)	0 (0)		1 (6.25)	0 (0)	
4	1 (1.75)	1 (1.9)		0 (0)	3 (5.2)		2 (12.5)	3 (5.7)	
5	1 (1.75)	1 (1.9)		0 (0)	0 (0)		2 (12.5)	4 (7.5)	
6 (Died)	2 (3.5)	1 (1.9)		1 (12.5)	9 (15.5)		2 (12.5)	5 (9.4)	

† median (range) days for all patients.

* p value for cross tabulation of all MRS categories.

Excluding death, the comAEM group had the highest (p = 0.008) proportion of patients with a residual disability (MRS = 1–5): 19/62 (30.6%) vs. 15/105 (14.3%, comBM) and 6/56 (10.7%, comTBM); most of the comAEM group had severe disability.

## Discussion

This prospective study has documented the range of pathogens causing CNS infections in patients admitted to a referral hospital in Hanoi. A confirmed diagnosis was made in just over a quarter of patients and most often in the patients with confirmed bacterial meningitis. When combining the confirmed and suspected diagnoses, bacterial meningitis remained the principal diagnostic group followed by TBM and AEM, findings consistent broadly with data from a referral hospital in HCM city. [15]

The low proportion of confirmed diagnoses is similar to other clinical series [14] [34]–[35] and is a consequence of previous antibiotic use, natural clearance of virus, targeting a preselected number of pathogens for PCR, late lumbar punctures [36], and the low sensitivity of diagnostic tools currently available for confirming TB. [37] Indeed, the CSF findings in the sBM group were significantly different to the cBM group and were consistent with partially treated meningitis.

The sensitivity of the Gram stain in our setting was low, ∼25% for comBM and ∼40% for cBM; other series have reported sensitivities of 60 to 90% in previously untreated patients [38]–[40] and 40 to 60% in partially treated meningitis. [41] Although a history of preadmission antibiotic use did not apparently affect our Gram stain results, according with some studies [42], [43] but not others [44], it certainly reduced our ability to confirm bacterial meningitis, a not unexpected finding. [43]–[46]

The reported sensitivity of ZN stain varies widely from lows of 5 [47] to 13% [48] to a high of 91%. [49] In a study in HCM city, the ZN stain sensitivity was 58% (77/132). Importantly, this study identified three practical aspects in obtaining a positive ZN stain, namely, the volume of CSF, proper concentration of CSF by centrifugation, and the time taken to read the slide. [50] Failing to detect any AFB suggests the possible need for further capacity enhancement in our laboratory and the need to encourage physicians to take an adequate CSF volume. A minimum of 6 mL of CSF for TB diagnosis alone is recommended and should be examined for at least 30 minutes. [50] In the past, CSF volumes of 10–20 mL were taken routinely with good results [49] and good tolerability by patients. [51] As the normal rate of CSF fluid formation in adults is ∼500 mL/day, there is no reason to collect insufficient volumes for proper microbiological diagnosis. [52]

The use of molecular analyses enhanced substantially our ability to diagnose patients. Indeed, PCR was the only diagnostic tool in our setting for viral pathogens. HSV and VZV were the two main viral pathogens detected and this diagnosis allowed appropriate treatment to be given. Enterovirus, an important paediatric pathogen, was uncommon in this adult population. We also tested CSF for presence of Nipah virus by RT-PCR in a subgroup of 81 patients with non-purulent meningitis but all were negative. We did not pursue dengue or JE viruses by examining the CSF for specific IgM antibodies. [53] Dengue is a well described but unusual cause of encephalitis [6] and JEV is confined mostly to children. [3], [23]

Similar to data from HCM city, we identified *S. suis* as the main cause of bacterial meningitis, a disease associated with occupational exposure to pigs or pork. [5] Eating undercooked pig products, a common practice in Vietnam, is also a risk factor. [54] Pneumococcal and meningococcal meningitis, frequent causes in many adult clinical series [34], [55], were uncommon in our patients and less frequently detected compared to patients in HCM city [9], for unknown reasons. We detected *S. pasteurianus* and *L. monocytogenes* by 16S rRNA analysis. The former has been proposed to replace *S. bovis* biotype 11/2 [56] and is a rare cause of adult meningitis. [57] We believe this is the first time it has been reported in Vietnam. Listeria is also a rare cause of meningitis in Vietnam. [10] In two developed Asian counties, listeria meningitis appears to be uncommon in Singapore [58] but accounted for just under 7% of adult meningitis in south Korea. [59]

Our study had limitations. This study took place at a referral hospital so our findings may not represent the epidemiology of CNS infections in the wider community. We had reasonable but limited microbiological diagnostic capacity; thus, some pathogens were probably missed. We combined confirmed and suspected diagnoses and analysed across these groups, cogniscent that diagnostic accuracy, most likely in our sBM group, suffers when relying on clinical features, the results of non specific investigations and “grey” CSF findings. [60]–[62] We did not seek unusual, non infectious causes of lymphocytic predominant CSF like collagen vascular disease, acute disseminated encephalomyelitis.

To conclude, this study has identified bacterial meningitis caused by *S. suis* as the main cause of CNS infections in adults at our referral hospital in Hanoi. PCR was particularly helpful for the early diagnosis and treatment in the AEM group. The overall low rate of confirmed infections calls for better diagnostic tests.
